# Brazilian Longitudinal Study of Adult Health (ELSA-Brasil) participant’s profile regarding self-rated health: a multiple correspondence analysis

**DOI:** 10.1186/s12889-021-11760-2

**Published:** 2021-09-27

**Authors:** Thaís Lopes de Oliveira, Raquel Vasconcellos Carvalhaes de Oliveira, Rosane Harter Griep, Arlinda B. Moreno, Maria da Conceição Chagas de Almeida, Ylva Brännström Almquist, Maria de Jesus Mendes da Fonseca

**Affiliations:** 1 Department of Epidemiology and Quantitative Methods in health. National School of Public Health, Oswaldo Cruz Foundation, 1480, Manguinhos, Rio de Janeiro, RJ 21041-210 Brazil; 2 Clinical Epidemiology Laboratory, Evandro Chagas National Institute of Infectious Diseases (INI), Oswaldo Cruz Foundation, Rio de Janeiro, Brazil; 3 Laboratory of Health and Environment Education, Oswaldo Cruz Institute, Oswaldo Cruz Foundation, Rio de Janeiro, Brazil; 4 Gonçalo Moniz Institute, Oswaldo Cruz Foundation, Salvador, Brazil; 5 Department of Public Health Sciences, Centre for Health Equity Studies (CHESS), Stockholm University, Stockholm, Sweden

**Keywords:** Self-rated health, Job strain, Multivariate analysis, Health inequality

## Abstract

**Background:**

Self-rated health (SRH) - one of the most common health indicators used to verify health conditions - can be influenced by several types of socioeconomic conditions, thereby reflecting health inequalities. This study aimed to evaluate the participant profiles regarding the association between self-rated health and social and occupational characteristics of the Brazilian Longitudinal Study of Adult Health (ELSA-Brasil).

**Methods:**

Cross-sectional design, including 11,305 individuals. Self-rated health was categorized as good, fair, and poor. The relationship between socio-demographic, psychosocial work environment, health-related variables, and self-rated health was analyzed by multiple correspondence analysis (stratified by age: up to 49 years old and 50 years old or more).

**Results:**

For both age strata, group composition was influenced by socioeconomic conditions. Poor SRH was related to lower socioeconomic conditions, being women, black self-declared race/ethnicity, being non-married/non-united, low decision authority, low skill discretion, and obesity.

**Conclusion:**

To promote health, interventions should focus on reducing existing socioeconomic, race, and gender inequalities in Brazil.

## Background

Socioeconomic conditions (such as education and income), basic sanitation, housing, nutrition, working conditions, as well as access to health services and information are some of the factors that affect the health of an population [1]. Research has shown that inequalities by race [2, 3], socioeconomic conditions [3], gender [3], gender in the workplace [3, 4], and by regions [5] are still a reality in Brazil, and these factors are strongly related to health [6–9]. The most common health indicators used to verify health conditions is self-rated health, and some studies show that these inequalities contribute to a poor state of health [7, 8, 10].

Along with these inequalities, the conditions in the workplace also have an important role in self-rated health. Several authors [7, 9, 11] demonstrated interesting results about how unemployment, informal work, job strain, high job demands, low job control, and an imbalance between work and social life influence negatively on self-rated health.

All of these factors are related in a complex way to self-rated health, and it is important to verify how these relationships are influenced by the existing inequalities in Brazil and how they are associated with health in the working population. Different methods have been applied to investigate negative perceptions of health [10, 12]. The large majority of studies have been using regression or multilevel models [8, 12, 13]. However, another way to examine this, which pays more attention to exploring and explaining relationships between categorical indicators, is through multiple correspondence analysis. The advantage of this statistical method is the absence of any assumption about probability distributions and the lack of need to establish predetermined relations among the variables.

Some studies show that the correspondence analysis is a technique that make it possible to illustrate the relationships between several categorical variables [14, 15], and also allows for the “construction of complex visual maps whose structuring can be interpreted” [16]. Thus far, we have found few studies envolving self-rated health that used correspondence analysis [15, 17, 18]. Accordingly, the aim of this study was to evaluate participant profiles regarding the association of self-rated health and social and occupational characteristics in the Brazilian Longitudinal Study of Adult Health (ELSA-Brasil), using the multiple correspondence analysis technique.

## Methods

### Study population

This study used baseline data (2008–2010) from the ELSA-Brasil study. The ELSA-Brasil is a longitudinal multicentric cohort study of 15,105 civil servants (35–74 years) conducted at six study research centres in three regions of the country, including the Northeast, South, and Southeast. These research centres are located in five federal universities and the Oswaldo Cruz Foundation [19, 20].

**Fig. 1:**
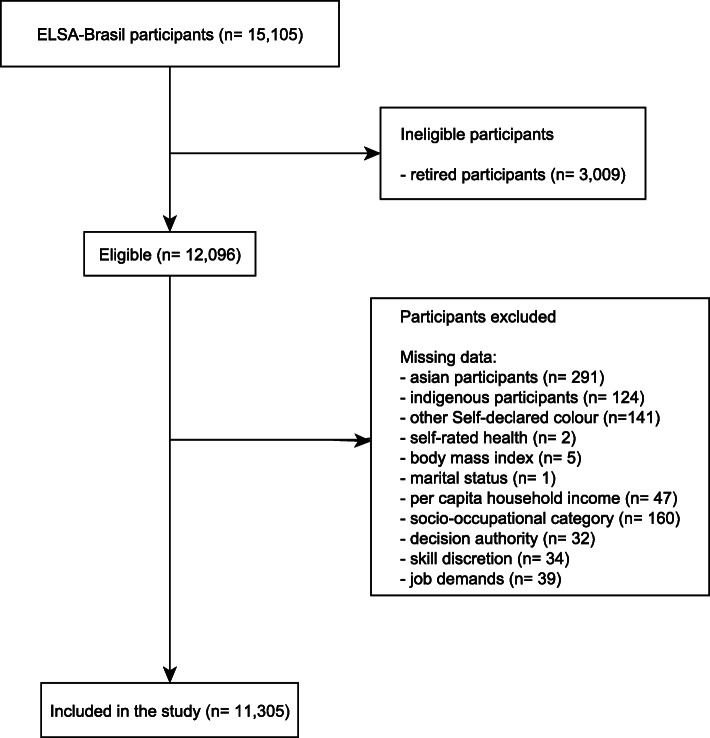
Flow-chart of participants, ELSA-Brasil, baseline data, 2008–2010

The present study did not use information about retired participants, since they do not have information about occupational characteristics (socio-occupational category and psychosocial work environment). Also, participants that declared their race/ethnicity as Asian or Indigenous were excluded due to the small number of participants in each category (2.4 and 1%, respectively). Furthermore, the information of participants that declared their race/ethnicity as Asian is mainly centered in one of the research centres in São Paulo. The exclusion of Indigenous people was made considering that our participants are urban indigenous in a small number, and they do not represent the indigenous population. Finally, participants who had missing data for any of the study variables were also excluded (Fig. 1).

### Socio-demographic variables

The variables were age, sex, self-declared race/ethnicity (white, brown and black), marital status (married/united, non-married/non-united – this category includes single, divorced or separated, and widowed people), education (the categories were complete elementary school or less, completed high school, and completed university degree or more. This variable considers the highest level of completed education, with exception of elementary school, i.e. a participant that did not completed high school was considered in the complete elementary school or less category), month per capita household income (low - up to $234, medium - from $234 to $702, and high - more than $702. The cutoff points of this variable were based on the 2008 minimum wage in Brazil and the median income of our population. The low category considers one salary, the medium category considers from 1 to 3 salaries, and the high category considers more than 3 salaries. The median income of our population was $702), and socio-occupational category (manual, middle and higher). This last variable considers different forms of insertion in production (considering the position in the typical occupation), the occupation itself (which non-manual was qualified by the level of formal education required by the occupation and manual was qualified by the sector specialization), and the hierarchy in production. The categories were defined by the Center for Development and Regional Planning (CEDEPLAR), Faculty of Economic Sciences of the Federal University of Minas Gerais (UFMG), based on the literature [21, 22].

### Psychosocial work environment variable

The variable representing the psychosocial work environment was job stress (demand–control model). Job stress was accessed using the Swedish demand control support questionnaire (DCSQ). This questionnaire contains 17 items, five items refer to the psychological demand dimension, six items refer to the control dimension, and six items refer to the social support dimension. In our study, the repetitive work item (control dimension) and the social support dimension were not considered since the study about the dimensional structure of the DCSQ in the Brazilian context suggests this item exclusion and a better goodness-of-fit without the social support dimension [23]. The scores of the DCSQ (job demands, 5 items; skill discretion, 3 items; and decision authority, 2 items) were dichotomized into high and low at the median for these dimensions (14, 11, and 6 points, respectively) [24].

### Health related variables

The health variables used in analyses were self-rated health and body mass index. Self-rated health (SRH) was measured using the following question: “*In general, compared to people of your age, how do you consider your state of health?*”. The response options were: “*very good, good, fair, poor, or very poor*”. For the analyses, the answers were categorized as good self-rated health (very good and good), fair, and poor (poor and very poor). The body mass index (BMI) cutpoints were considered as: ≤ 24.9 kg/m^2^ for underweight and normal weight (the categories of underweight, ≤ 18.5 kg/m^2^, and normal weight were grouped due to the small number of participants who were underweight, < 1%), between 25 and 29.9 kg/m^2^ for overweight, and ≥ 30 kg/m^2^ for obesity.

### Statistical analyses

Proportions were used to describe population characteristics regarding self-rated health. Self-rated health, sex, self-declared race/ethnicity, marital status, education, per capita household income, body mass index, socio-occupational category, and job strain were analyzed by multiple correspondence analysis (MCA). Stratified analyses by age were conducted due to our consideration of aging as an effect modifier [7, 12, 25]. Since our average population age is 49.14 years, two age groups were created to stratify the analyses (up to 49 years old and 50 years old or more).

Correspondence analysis is an exploratory technique applied to categorical data. This analysis graphically illustrates the relationship within one set of variables, and the proximity of categories, in space, indicates a relationship or correspondence between them [26, 27]. The advantage of this statistical method is the absence of any assumption about probability distributions and the lack of a need to establish predetermined relations among the variables, such as the unidirectional relationships estimated by regression models. This type of analysis provides total inertia, which means the percentage of variability explained by each dimension, and in this paper, the number of dimensions was chosen by analyzing the decline of adjusted inertias (eigenvalues) [27].

Scatterplots (formed by the coordinates of each category in each dimension) were analyzed with regard to dimensions, and clusters of categories were created to delineate different profiles in the sample. The results based on hierarchical cluster analysis (dendrogram) of the standard coordinates obtained in the correspondence analysis were confronted with the resulting clusters visualized in the multiple correspondence plot. The dendrogram provided a clear visualization of the categories of the variables in each group, and it is more useful in the case of many dimensions in the MCA (which become hard to use biplots).

The x-axis of the scatterplots represents the data variability explained by the first dimension, while the y-axis represents the data variability explained by the second dimension. The dots represent each variables categories.

The analyses were performed in the R software [28], version 3.5.1, library “ca”, “ggplot2”, “dendextend”, and “factoextra”.

## Results

In the study population (11,305 participants), the largest proportion of individuals reported good SRH (81.6%), followed by fair (16.7%) and poor (1.7%) categories. The percentage of poor self-rated health was lower among men, married/united, white self-declared race/ethnicity, participants aging up to 49 years old, with completed university degree or more, with high per capita household income, with higher socio-occupational category, with normal weight, with high job demands, high skill discretion, and high decision authority (Table 1).

**Table 1:** Distribution of study variables by self-rated health of 11,305 civil servants, ELSA-Brasil, baseline data, 2008–2010

	Self-rated health		
	Good	Fair	Poor
	*n* = 9221 (%)	*n* = 1889 (%)	*n* = 195 (%)
Sex			
Women	4811 (81.6)	960 (16.3)	126 (2.1)
Men	4410 (81.5)	929 (17.2)	69 (1.3)
Age			
up to 49 years old	5112 (84.4)	861 (14.2)	85 (1.4)
50 years old or more	4109 (78.3)	1028 (19.6)	110 (2.1)
Self-declared race/ethnicity			
black	1443 (75)	431 (22.5)	49 (2.5)
brown	2687 (78.2)	679 (19.8)	70 (2)
white	5091 (85.6)	779 (13.1)	76 (1.3)
Marital status			
married/united	6265 (81.8)	1277 (16.6)	119 (1.6)
non-married/non-united	2956 (81.1)	612 (16.8)	76 (2.1)
Education			
complete elementary school or less	764 (62)	424 (34.4)	45 (3.6)
completed high school	3252 (78.1)	822 (19.7)	89 (2.2)
completed university degree or more	5205 (88.1)	643 (10.9)	61 (1)
Per capita household income			
low	932 (68.1)	389 (28.4)	48 (3.5)
medium	3401 (78.4)	845 (19.5)	94 (2.1)
high	4888 (87.4)	655 (11.7)	53 (0.9)
Socio-occupational category			
higher	3625 (87.7)	457 (11.1)	51 (1.2)
middle	4117 (81.3)	862 (17)	88 (1.7)
manual	1479 (70.2)	570 (27.1)	56 (2.7)
BMI			
normal weight	3727 (87.6)	479 (11.3)	45 (1.1)
overweight	3716 (82.3)	742 (16.4)	57 (1.3)
obesity	1778 (70)	668 (26.3)	93 (3.7)
Job demands			
high	3395 (81.3)	712 (17)	70 (1.7)
low	5826 (81.7)	1177 (16.5)	125 (1.8)
Skill discretion			
high	3807 (85.9)	570 (12.9)	55 (1.2)
low	5414 (78.8)	1319 (19.2)	140 (2)
Decision authority			
high	3395 (84.5)	565 (14.1)	57 (1.4)
low	5826 (79.9)	1324 (18.2)	138 (1.9)

**Fig. 2:**
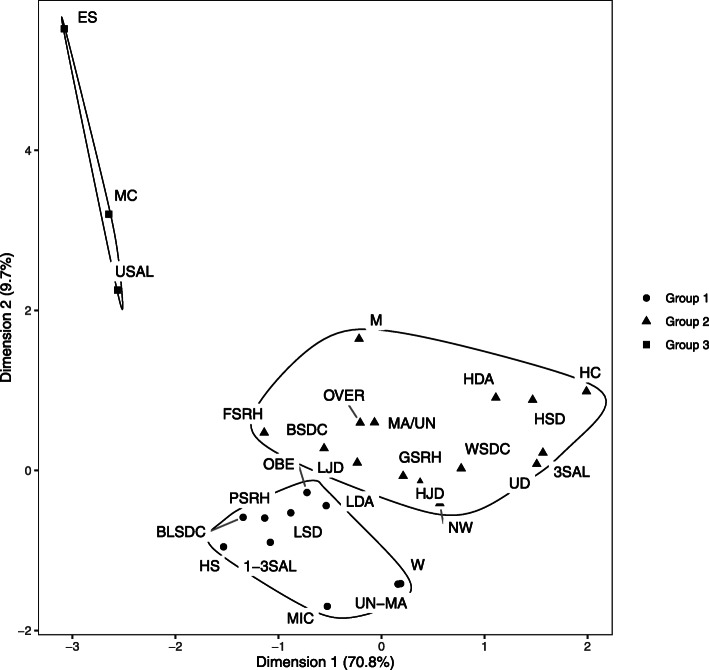
Two dimension plot of multiple correspondence among participants with up to 49 years old, ELSA-Brasil, 2008–2010. W: women; M: men;BLSDC: Black self-declared race/ethnicity; BSDC: Brown self-declared race/ethnicity; WSDC: White self-declared race/ethnicity; MA/UN: Married/united; UN-MA: Non-married/non-united; ES: complete elementary school or less; HS: Completed high school; UD: Completed university degree or more; USAL: Up to a salary; 1-3SAL: From 1 to 3 salaries;3SAL: More than 3 salaries; MC: Manual category; MIC: Middle category; HC: Higher category; HAD: High decision authority; LDA: Low decision authority; HJD: High job demands; LJD: Low job demands; HSD: High skill discretion; LSD: Low skill discretion; GSRH: Good SRH; FSRH: Fair SRH; PSRH: Poor SRH; NW: Normal weight; OVER: Overweigh; OBE: Obesity

**Fig. 3:**
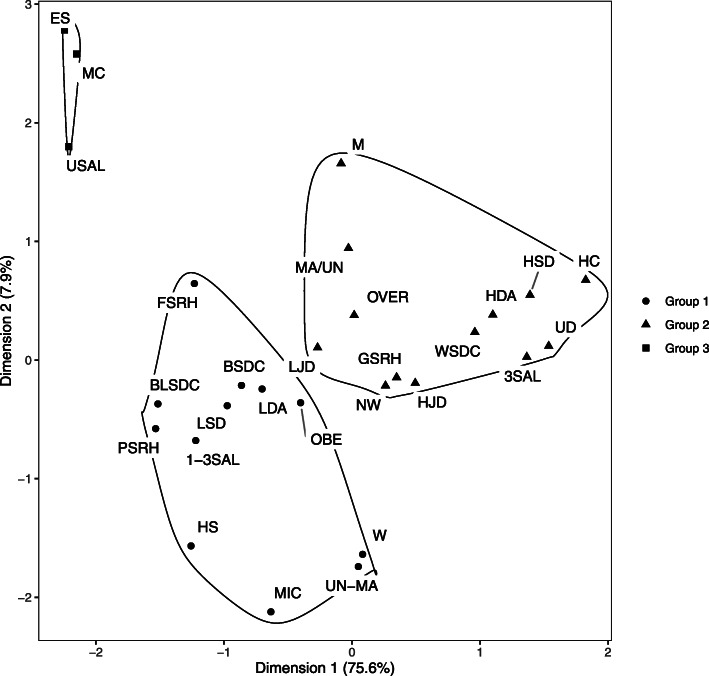
Two dimension plot of multiple correspondence among participants with 50 years old or more, ELSA-Brasil, 2008–2010. W: women; M: men;BLSDC: Black self-declared race/ethnicity; BSDC: Brown self-declared race/ethnicity; WSDC: White self-declared race/ethnicity; MA/UN: Married/united; UN-MA: Non-married/non-united; ES: complete elementary school or less; HS: Completed high school; UD: Completed university degree or more; USAL: Up to a salary; 1-3SAL: From 1 to 3 salaries; 3SAL: More than 3 salaries; MC: Manual category; MIC: Middle category; HC: Higher category; HAD: High decision authority; LDA: Low decision authority; HJD: High job demands; LJD: Low job demands; HSD: High skill discretion; LSD: Low skill discretion; GSRH: Good SRH; FSRH: Fair SRH; PSRH: Poor SRH; NW: Normal weight; OVER: Overweigh; OBE: Obesity

The multiple correspondence analyses were stratified by age. The plot allowed for the identification of three groups for both age strata (Figs. 2 and 3). Figure 2 presents the plot of multiple correspondence analysis for participants up to 49 years old, and Fig. 3 for participants 50 years old or more. For the youngest group (up to 49 years old), the inertia of the two first dimensions was 80.5%. The first dimension explained 70.8% of data variability (x-axis of the graph) and the second 9.7% (y-axis of the graph). For the oldest group, the inertia of the two first dimensions was 83.5%. The first dimension explained 75.6% of data variability (x-axis of the graph) and the second 7.9% (y-axis of the graph).

The MCA results show modest, but relevant, differences between age groups. Figure 2 (up to 49 years old) shows that fair SRH and brown self-declared race/ethnicity were in the same group as better socio-economic conditions and good SRH. Figure 3 (50 years old or more) shows the same categories related to middle socio-economic conditions and poor SRH.

Besides fair SRH and brown self-declared race/ethnicity, there was no difference in the MCA results between the two age strata. Men, white self-declared race/ethnicity, married/united, completed university degree or more, higher socio-occupational category, high per capita household income, high decision authority, high skill discretion, high and low job demands, normal weight, and overweight were related to good SRH. Women, black self-declared race/ethnicity, non-married/non-united, completed high school, middle socio-occupational category, medium per capita household income, low decision authority, low skill discretion, and obesity were related to poor SRH. One of the cluster groups was not related to SRH. This group (group 3 for both age strata) included participants with complete elementary school or less, low per capita household income, and manual socio-occupational category (Figs. 2 and 3).

## Discussion

In this study, multiple correspondence analysis was used as a way to graphically represent and interpret the relationship between self-rated health and social and occupational characteristics. The composition of the different groups formed in the MCA reflected existing socioeconomic inequalities in Brazil. The results, similar for both age strata, show groups divided by better (group 2), average (group 1), and worse (group 3) socioeconomic conditions. The group with better conditions was related to good SRH, white self-declared race/ethnicity, and being men. Meanwhile, the average socioeconomic group was associated with poor SRH, black self-declared race/ethnicity, and being women. Lastly, the worst socioeconomic group was not related to social characteristics.

Since 2001, several policies have been presented by the Brazilian government [1, 29], focused on increasing the educational level [30], equalizing the income distribution [29], reducing poverty [29], and improving access to health services [6]. Despite these advances, Brazil is still a country burdened by inequalities [4, 6, 31]. After 2016, socioeconomic inequality begun to increase again, and the “*Continuous National Household Sample Survey (PNAD-contínua)*” shows differences in average earnings according to levels of education [32], and important racial disparities in education, employment, and income between white and non-white population (black and brown) [2].

The ELSA-Brasil is composed of civil servants of higher education institutions, with a career path, in which occupations require a certain level of education. As expected, the MCA results show an association between education and socio-occupation category. Even with these population characteristics, the results of the current study add to the knowledge about working conditions and socioeconomic, racial, and gender inequalities in Brazil. In our study, low decision authority at work and low skill discretion were related to being women, black self-declared race/ethnicity, average socioeconomic conditions, and middle socio-occupational category.

Despite the actions by the Brazilian government to promote gender equality in the workplace [33] and increase women’s access to education [30], Brazil [34] had the worst percentage of women in politics position (10,5%) among South American countries, and women with the same years of study and occupation as men, still receive lower wage [4, 31]. Also, in Brazil, the non-white population (black and brown) have lower education, and when employed, they usually received half of the income that white population received [2].

Another Brazilian study with civil servants found similar results. Women had more job strain and psychological distress than men. However, occupational status did not have the same role in psychological distress for both genders, as men with routine-non-manual or manual work had a higher prevalence [35].

Independent of these differences, several occupational studies demonstrated the importance of working conditions for health [7, 9, 11]. Brazilian studies with the working population demonstrated an association between job strain and cardiovascular risk [36], metabolic syndrome [37], migraine [38], poor quality of life [39], poor self-rated health [40], job dissatisfaction [41], and sickness-absenteeism from the job [42]. Our study also adds to the knowledge about working and health conditions since low decision authority at work, and low skill discretion were related to obesity and poor self-rated health. The prevalence of obesity has been increasing over the years in Brazil [43], and during our baseline the prevalence increased from 13.4% in 2008 to 14.9% in 2010 [44]. Some studies have shown that high values of body mass index are associated with poor self-rated health [45, 46], and despite the unclear relationship between job strain and the development of obesity [47], some longitudinal studies found an association between changes in BMI [48], abdominal obesity [49], and job strain.

Our results also show a small difference between “up to 49 years old” and “50 years old or more” groups’ composition. In the oldest group, fair SRH and brown self-declared race/ethnicitywere associated with poor SRH. Aging is pointed out as an important condition for the deterioration of health over the years [12, 13, 25] even after adjustment for socioeconomic conditions (income, education, and occupation) [25], and aging itself is a possible explanation for older people considering that fair SRH is in the same group as poor. This result shows that self-rated health should perhaps not always be stratified as poor or good, as our study shows that fair SRH may represent different conditions depending on age.

Finally, our study had similar results to other international studies [50–52]. Better education, income, and socio-occupational category were related to good self-rated health. These results reinforce that self-rated health is a relevant indicator to analyse health conditions in different countries with different social backgrounds.

One of the limitations of the present study is the generalization of our findings to the non-worker population, as our results are from a cohort of civil servants. However, one of the advantages of this study was the possibility to describe the complex relationship between self-rated health and occupational characteristics, since correspondence analysis is a technique to explain these relationships. A limitation of this type of analysis consists of being an exploratory technique that provide only point estimates. However, this limitation allowed the participants’ profiles identification without the results being affected by our sample size, which minor effects could lead to statistically significant tests [53]. In this way, the group composition of this study could be used in future studies that consider longitudinal analysis in a working population. Another limitation of our study is the exclusion of 3,3% of our sample due to missing values. However, these missing values did not differ between socio-demographic characteristics.

## Conclusions

To conclude, our study reinforces the relevance of the non-dichotomization of self-rated health. The results show that our participant profiles regarding self-rated health are similar for both age groups, and existing gender, racial, socioeconomic, and workplace inequalities somehow affected the group compositions. It was also possible to observe the importance of the psychosocial work environment on self-rated health and obesity, suggesting that further longitudinal studies are necessary to understand the relationship between these health conditions and occupational characteristics. In this way, in addition to health promotion policies, more actions need to be done to continue reducing inequalities in Brazil, since it may have an important role in health conditions.

## Data Availability

The public access to the database is closed. The database used and analysed during the current study are available from the corresponding author on reasonable request on the link http://www.elsa.org.br/contatos.html.

## Abbreviations

ELSA-Brasil: Brazilian Longitudinal Study of Adult Health
SRH: Self-rated health
BMI: Body mass index
MCA: Multiple correspondence analysis
*PNAD-contínua*: *Continuous National Household Sample Survey*
CAPES: Coordination for the Improvement of Higher Education Personnel

## References

[1] INCA IN do Câncer. Abordagem e Tratamento do Fumante - Consenso. Rio de Janeiro: Ministério da Saúde. 2001. p. 38.

[2] BRASIL. Instituto Brasileiro de Geografia e Estatística. PNAD Contínua 2016: 51% da população com 25 anos ou mais do Brasil possuíam no máximo o ensino fundamental completo. 2017. [cited 2020 Apr 1]. Available from: https://agenciadenoticias.ibge.gov.br/agencia-sala-de-imprensa/2013-agencia-de-noticias/releases/18992-pnad-continua-2016-51-da-populacao-com-25-anos-ou-mais-do-brasil-possuiam-no-maximo-o-ensino-fundamental-completo.

[3] BRASIL. Instituto Brasileiro de Geografia e Estatística. Coordenação de População e Indicadores Sociais. Síntese de indicadores sociais: uma análise das condições de vida da população brasileira. 2018. [cited 2019 Feb 22]. Available from: https://biblioteca.ibge.gov.br/visualizacao/livros/liv101629.pdf.

[4] BRASIL. Estatísticas de Gênero. Indicadores sociais das mulheres no Brasil. 2019. [cited 2019 Jul 15]. Available from: https://biblioteca.ibge.gov.br/visualizacao/livros/liv101551_informativo.pdf.

[5] Viacava F, Porto SM, Carvalho C de C, Bellido JG. Desigualdades regionais e sociais em saúde segundo inquéritos domiciliares (Brasil, 1998–2013). 2019;16. 10.1590/1413-81232018247.15812017 31340291

[6] Albuquerque MV (2017). de, Viana AL d’Ávila, Lima LD de, Ferreira MP, Fusaro ER, Iozzi FL. Desigualdades regionais na saúde: mudanças observadas no Brasil de 2000 a 2016. Ciênc saúde coletiva.

[7] Andrade FCD, Wu F, An R, Stellrecht A (2016). Employment status and health outcomes among Brazilian adults. Int Health.

[8] Szwarcwald CL, Damacena GN, Souza Júnior PRB (2015). de, Almeida W da S de, Lima LTM de, Malta DC, et al. Determinantes da autoavaliação de saúde no Brasil e a influência dos comportamentos saudáveis: resultados da Pesquisa Nacional de Saúde, 2013. Revista Brasileira de Epidemiologia.

[9] Milner A, Witt K, Spittal MJ, Bismark M, Graham M, LaMontagne AD. The relationship between working conditions and self-rated health among medical doctors: evidence from seven waves of the Medicine In Australia Balancing Employment and Life (Mabel) survey. BMC Health Serv Res. 2017;17(1):609. Available from: http://bmchealthservres.biomedcentral.com/articles/10.1186/s12913-017-2554-z. 10.1186/s12913-017-2554-z PMC5576303 28851354

[10] Santos SM, Werneck GL, Faerstein E, Lopes CS, Chor D. Focusing neighborhood context and self-rated health in the Pró-Saúde Study. Cadernos de Saúde Pública. 2018;34(5). Available from: http://www.scielo.br/scielo.php?script=sci_arttext&pid=S0102-311X2018000505017&lng=en&tlng=en. 10.1590/0102-311x00029517 29846405

[11] Giatti L, Barreto SM, César CC (2010). Unemployment and self-rated health: neighborhood influence. Soc Sci Med.

[12] Abebe DS, Tøge AG, Dahl E. Individual-level changes in self-rated health before and during the economic crisis in Europe. Int J Equity Health. 2016;15(1). Available from: http://www.equityhealthj.com/content/15/1/1. 10.1186/s12939-015-0290-8 PMC4700771 26728322

[13] Andrade FCD, Mehta JD. Increasing educational inequalities in self-rated health in Brazil, 1998–2013. Abe T, editor. PLOS ONE. 2018;13(4):e0196494. 10.1371/journal.pone.0196494 PMC5927445 29708990

[14] Sourial N, Wolfson C, Zhu B, Quail J, Fletcher J, Karunananthan S, Bandeen-Roche K, Béland F, Bergman H (2010). Correspondence analysis is a useful tool to uncover the relationships among categorical variables. J Clin Epidemiol.

[15] Meneguci J, Sasaki JE, da Silva SÁ, Scatena LM, Damião R (2015). Socio-demographic, clinical and health behavior correlates of sitting time in older adults. BMC Public Health.

[16] Ayele D, Zewotir T, Mwambi H (2015). Multiple correspondence analysis as a tool for analysis of large health surveys in African settings. Afr Health Sci.

[17] Veenstra G (2007). Social space, social class and Bourdieu: health inequalities in British Columbia, Canada. Health & Place.

[18] Burnett PJ, Veenstra G (2017). Margins of freedom: a field-theoretic approach to class-based health dispositions and practices. Sociol Health Illn.

[19] Schmidt MI, Duncan BB, Mill JG, Lotufo PA, Chor D, Barreto SM, Aquino EM, Passos VMA, Matos SM, Molina MCB, Carvalho MS, Bensenor IM (2015). Cohort profile: longitudinal study of adult health (ELSA-Brasil). Int J Epidemiol.

[20] Aquino EML, Barreto SM, Bensenor IM, Carvalho MS, Chor D, Duncan BB, Lotufo PA, Mill JG, Molina MDC, Mota ELA, Azeredo Passos VM, Schmidt MI, Szklo M (2012). Brazilian longitudinal study of adult health (ELSA-Brasil): objectives and design. Am J Epidemiol.

[21] Machado AF, Oliveira AMHC. Tipologias Ocupacionais aplicadas à análise socioeconômica da amostra Elsa (1a onda). Relatório Técnico Projeto ELSA. CEDEPLAR, UFMG; 2013.

[22] Faleiro JC, Giatti L, Barreto SM, Camelo L do V, Griep RH, Guimarães JMN, et al. Posição socioeconômica no curso de vida e comportamentos de risco relacionados à saúde: ELSA-Brasil. Cad Saúde Pública. 2017. [cited 2021 17]. Available from: http://www.scielo.br/scielo.php?script=sci_arttext&pid=S0102-311X2017000305005&lng=pt&tlng=pt. 10.1590/0102-311X00017916 28380138

[23] Hökerberg YHM, Aguiar OB, Reichenheim M, Faerstein E, Valente JG, Fonseca M de J, et al. Dimensional structure of the demand control support questionnaire: a Brazilian context. Int Arch Occup Environ Health. 2010;83(4):407–16. 10.1007/s00420-009-0488-4. 10.1007/s00420-009-0488-4 19941002

[24] Alves MG (2004). de M, Chor D, Faerstein E, Lopes C de S, Werneck GL. Versão resumida da ‘job stress scale’: adaptação para o português. Rev Saude Publica.

[25] Cullati S. Socioeconomic inequalities in health trajectories in Switzerland: are trajectories diverging as people age?. Sociol Health Illn. 2015;37(5):745–64. 10.1111/1467-9566.12232. 10.1111/1467-9566.12232 25683678

[26] Greenacre M, Blasius J (2006). Multiple correspondence analysis and related methods.

[27] Paula F de L, Fonseca M de JM da, Oliveira R de VC de, Rozenfeld S. Perfil de idosos com internação por quedas nos hospitais públicos de Niterói (RJ). Rev bras epidemiol. 2010;13(4):587–595. 10.1590/S1415-790X2010000400004. 10.1590/s1415-790x2010000400004 21180848

[28] R Core Team. R: A language and environment for statistical computing. Vienna: R Foundation for Statistical Computing; 2018. Available from: https://www.R-project.org/.

[29] BRASIL. Instituto de Pesquisa Econômica Aplicada. Programa Bolsa Família: uma década de inclusão e cidadania: Sumário executivo. 2014. Available from: http://www.mds.gov.br/webarquivos/publicacao/bolsa_familia/Livros/Bolsa10anos_Sumex_Port.pdf.

[30] Beltrão KI, Alves JED (2009). A reversão do hiato de gênero na educação brasileira no século XX. Cad Pesqui.

[31] Bruschini MCA (2007). Trabalho e gênero no Brasil nos últimos dez anos. Cad Pesqui.

[32] BRASIL. Intituto Brasileiro de Geografia e Estatística. Continuous PNAD 2018: 10% of population concentrate 43.1% of Brazilian wage bill. 2018. [cited 2020 Apr 1]. Available from: https://agenciadenoticias.ibge.gov.br/en/agencia-press-room/2185-news-agency/releases-en/25706-continuous-pnad-2018-10-of-population-concentrate-43-1-of-brazilian-wage-bill.

[33] BRASIL. Instituto de Pesquisa Econômica Aplicada. Programa Pró-Equidade de Gênero e Raça. 2005. Available from: http://www.ipea.gov.br/sites/proequidade/o-que-e.

[34] BRASIL. Instituto Brasileiro de Geografia e Estatística. Gender Statistics: household chores affect insertion of women in labor market. 2018. Available from: https://agenciadenoticias.ibge.gov.br/en/agencia-press-room/2185-news-agency/releases-en/20262-gender-statistics-household-chores-affect-insertion-of-women-in-labor-market.

[35] Lopes CS, Araya R, Werneck GL, Chor D, Faerstein E (2010). Job strain and other work conditions: relationships with psychological distress among civil servants in Rio de Janeiro. Brazil Soc Psychiat Epidemiol.

[36] Marçal Pimenta A, Kac G (2012). Campos e Souza RR, Barros Almeida Ferreira LM de, de Fátima Silqueira SM. Trabalho noturno e risco cardiovascular em funcionários de universidade pública. Revista da Associação Médica Brasileira.

[37] Santos AE, Araújo LF, Griep RH, Castro Moreno CR, Chor D, Barreto SM, Giatti L (2018). Shift work, job strain, and metabolic syndrome: cross-sectional analysis of ELSA-Brasil. Am J Ind Med.

[38] Santos IS, Griep RH, Alves MGM, Goulart AC, Lotufo PA, Barreto SM, Chor D, Benseñor IM (2014). Job stress is associated with migraine in current workers: the Brazilian longitudinal study of adult health (ELSA-Brasil): job stress and migraine in current workers. EJP..

[39] Silva LS, Barreto SM (2012). Adverse psychosocial working conditions and poor quality of life among financial service employees in Brazil. Jrnl of Occup Health.

[40] Theme Filha MM, Costa MA de S, Guilam MCR. Estresse ocupacional e autoavaliação de saúde entre profissionais de enfermagem. 2013;1–9.

[41] de Sousa CC, de Araújo TM, Lua I, Gomes MR (2019). Occupational stress and job dissatisfaction with health work. Psicol Refl Crít.

[42] dos Santos K, Kupek E, Cunha JCCB, Blank VLG. Absenteísmo-doença, modelo demanda-controle e suporte social: um estudo caso-controle aninhado em uma coorte de trabalhadores de hospitais, Santa Catarina, Brasil. Rev Bras Epidemiol. 2011;14(4):609–19. 10.1590/S1415-790X2011000400008.

[43] BRASIL. Vigitel Brasil 2017: vigilância de fatores de risco e proteção para doenças crônicas por inquérito telefônico: estimativas sobre frequência e distribuição sociodemográfica de fatores de risco e proteção para doenças crônicas nas capitais dos 26 estados brasileiros e no Distrito Federal em 2017. Ministério da Saúde. Secretaria de Vigilância em Saúde. Departamento de Vigilância de Doenças e Agravos não Transmissíveis e Promoção da Saúde. 2018. Available from: http://bvsms.saude.gov.br/bvs/publicacoes/vigitel_brasil_2017_vigilancia_fatores_risco.pdf.

[44] Malta DC, Andrade SC, Claro RM, Bernal RTI, Monteiro CA. Trends in prevalence of overweight and obesity in adults in 26 Brazilian state capitals and the Federal District from 2006 to 2012. Rev Bras Epidemiol. 2014;17(suppl 1):267–76. 10.1590/1809-4503201400050021. 10.1590/1809-4503201400050021 25054269

[45] Wang A, Arah OA. Body Mass Index and Poor Self-Rated Health in 49 Low-Income and Middle-Income Countries, By Sex, 2002–2004. Prev Chronic Dis. 2015;12. Available from: http://www.cdc.gov/pcd/issues/2015/15_0070.htm. 10.5888/pcd12.150070 PMC4556100 26292064

[46] Cullinan J, Gillespie P (2016). Does overweight and obesity impact on self-rated health? Evidence using instrumental variables ordered Probit models: the impact of overweight and obesity on self-rated health. Health Econ.

[47] Kivimäki M, Singh-Manoux A, Nyberg S, Jokela M, Virtanen M (2015). Job strain and risk of obesity: systematic review and meta-analysis of cohort studies. Int J Obes.

[48] Fujishiro K, Lividoti Hibert E, Schernhammer E, Rich-Edwards JW (2017). Shift work, job strain and changes in the body mass index among women: a prospective study. Occup Environ Med.

[49] Ishizaki M, Nakagawa H, Morikawa Y, Honda R, Yamada Y, Kawakami N, The Japan Work Stress and Health Cohort Study Group (2008). Influence of job strain on changes in body mass index and waist circumference—6-year longitudinal study. Scand J Work Environ Health.

[50] Borg V, Kristensen TS (2000). Social class and self-rated health: can the gradient be explained by differences in life style or work environment?. Soc Sci.

[51] McFadden E, Luben R, Bingham S, Wareham N, Kinmonth A-L, Khaw K-T. Social inequalities in self-rated health by age: Cross-sectional study of 22 457 middle-aged men and women. BMC Public Health. 2008;8(1). [cited 2018 Nov 2]. Available from: http://bmcpublichealth.biomedcentral.com/articles/10.1186/1471-2458-8-230. 10.1186/1471-2458-8-230 PMC2491612 18611263

[52] Hosseinpoor AR, Stewart Williams J, Amin A, Araujo de Carvalho I, Beard J, Boerma T, et al. Social Determinants of Self-Reported Health in Women and Men: Understanding the Role of Gender in Population Health. Shea BJ, editor. PLoS ONE. 2012;7(4):e34799. 10.1371/journal.pone.0034799 PMC3326052 22514667

[53] Greenland S, Senn SJ, Rothman KJ, Carlin JB, Poole C, Goodman SN, Altman DG (2016). Statistical tests, P values, confidence intervals, and power: a guide to misinterpretations. Eur J Epidemiol.

